# Positive association between stress hyperglycemia ratio and pulmonary infection in patients with ST-segment elevation myocardial infarction undergoing percutaneous coronary intervention

**DOI:** 10.1186/s12933-023-01799-3

**Published:** 2023-03-31

**Authors:** Zehuo Lin, Xueqing Liang, Yeshen Zhang, Yining Dai, Lin Zeng, Weikun Chen, Siyu Kong, Pengcheng He, Chongyang Duan, Yuanhui Liu

**Affiliations:** 1 Department of Cardiology, Guangdong Provincial People’s Hospital (Guangdong Academy of Medical Sciences), Southern Medical University, Guangzhou, 510080 China; 2 Shantou University Medical College, Shantou, China; 3 Department of Biostatistics, School of Public Health, Southern Medical University, Guangzhou, China; 4 Department of Cardiology, Heyuan People’s Hospital, Heyuan, China

**Keywords:** ST-segment elevation myocardial infarction, Percutaneous intervention, Stress hyperglycemia ratio, Pulmonary infection, Risk factor

## Abstract

**Background:**

Previous studies have shown that the stress hyperglycemia ratio (SHR), a parameter of relative stress-induced hyperglycemia, is an excellent predictive factor for all-cause mortality and major adverse cardiovascular events (MACEs) among patients with ST-segment elevation myocardial infarction (STEMI). However, its association with pulmonary infection in patients with STEMI during hospitalization remains unclear.

**Methods:**

Patients with STEMI undergoing percutaneous coronary intervention (PCI) were consecutively enrolled from 2010 to 2020. The primary endpoint was the occurrence of pulmonary infection during hospitalization, and the secondary endpoint was in-hospital MACEs, composed of all-cause mortality, stroke, target vessel revascularization, or recurrent myocardial infarction.

**Results:**

A total of 2,841 patients were finally included, with 323 (11.4%) developing pulmonary infection and 165 (5.8%) developing in-hospital MACEs. The patients were divided into three groups according to SHR tertiles. A higher SHR was associated with a higher rate of pulmonary infection during hospitalization (8.1%, 9.9%, and 18.0%, P < 0.001) and in-hospital MACEs (3.7%, 5.1%, and 8.6%, P < 0.001). Multivariate logistic regression analysis demonstrated that SHR was significantly associated with the risk of pulmonary infection during hospitalization (odds ratio [OR] = 1.46, 95% confidence interval [CI] 1.06–2.02, P = 0.021) and in-hospital MACEs (OR = 1.67, 95% CI 1.17–2.39, P = 0.005) after adjusting for potential confounding factors. The cubic spline models demonstrated no significant non-linear relationship between SHR and pulmonary infection (P = 0.210) and MACEs (P = 0.743). In receiver operating characteristic curve, the best cutoff value of SHR for pulmonary infection was 1.073.

**Conclusions:**

The SHR is independently associated with the risk of pulmonary infection during hospitalization and in-hospital MACEs for patients with STEMI undergoing PCI.

**Supplementary Information:**

The online version contains supplementary material available at 10.1186/s12933-023-01799-3.

## Introduction

Infection during hospitalization, especially pulmonary infection, is a serious complication observed among patients with ST-segment elevation myocardial infarction (STEMI), which induces a tenfold increase in the 30-day mortality rate [1–4]. Early identification and intervention improve the effectiveness of treatment and outcomes.

Stress-induced hyperglycemia (SIH), defined as temporarily increased acute blood glycemia during an emergency situation, is a common state among patients with STEMI [5]. SIH is linked to infection both in vitro and in vivo [6, 7], and results in an increased release of proinflammatory cytokines [8, 9]. Prior studies have used admission blood glucose (ABG) as a parameter of stress hyperglycemia [10, 11]. However, the ABG level is influenced not only by acute stress but also by a chronic glycemic condition, which limits its ability to distinguish a true acute glycemic rise [12]. In recent years, the stress hyperglycemia ratio (SHR), calculated from the ABG adjusted by the average glycemic status [13], has been regarded as a better index of relative SIH compared to ABG alone [12]. The SHR has been reported as a reliable predictor for poor long- and short-term prognosis in patients with STEMI [12, 14, 15], and for stroke-associated pneumonia in patients without diabetes mellitus (DM) [16]. However, the prognostic value of the SHR for pulmonary infection during hospitalization among patients with STEMI remains unclear.

Therefore, the main aim of the present study was to investigate the association between SHR and pulmonary infection during hospitalization among patients with STEMI.

## Method

### Study population

Patients with STEMI at Guangdong Provincial People’s Hospital were consecutively enrolled between 2010 and 2020. Patients were diagnosed with STEMI according to the international guideline as follows: (1) typical chest pain or ischemia symptoms; (2) dynamic changes in the new electrocardiogram: ST-segment elevation of more than two adjacent leads or new left bundle branch block; (3) increased levels of myocardial-injury biochemical markers [17]. We excluded patients who (1) undergoing coronary artery bypass grafting, (2) with infection before the diagnosis of STEMI, (3) were on hemodialysis at admission, (4) died within 24 h after admission, (5) readmission to hospital, (6) did not receive the percutaneous coronary intervention (PCI) and (7) were missing crucial laboratory data (ABG or glycosylated hemoglobin [HbA1c]). This study was approved by the research ethics committee of Guangdong Provincial People’s Hospital and was conducted according to the guidelines stipulated in the Declaration of Helsinki.

### Data collection and definitions

Baseline blood samples were collected within 24 h of admission and tested for white blood cell count, hemoglobin, ABG, HbA1c, troponin I/T, cardiac enzymes, serum creatinine, blood lipids, and other routine blood tests. Relevant data were collected, including demographic information, medical history (hypertension, DM, myocardial infarction [MI] or coronary revascularization, stroke), admission status (heart rate, systolic and diastolic blood pressure, and Killip class), and coronary revascularization data. Killip class is used to assess the severity of acute myocardial infarction. Killip class I was defined as absence of congestive heart failure, class II as presence of rales and/or jugular venous distension, class III as presence of pulmonary oedema and class IV as cardiogenic shock. DM was defined based on the history of DM or newly diagnosed DM with HbA1c ≥ 6.5%, fasting blood glucose ≥ 7.0 mmol/L, or 2-h plasma glucose ≥ 11.1 mmol/L in the oral glucose tolerance test [18]. The SHR was defined as the index calculated by the formula: [(ABG (mg/dl))/(28.7 × HbA1c (%) − 46.7)] [13].

Coronary angiography and/or PCI were performed at the interventional cardiologists’ discretion and according to the current guidelines [19]. Medications, including aspirin, clopidogrel or ticagrelor, angiotensin-converting enzyme inhibitors or angiotensin receptor blockers, and β-blockers, were prescribed by the cardiologists according to current guidelines and recommendations. Transthoracic echocardiography, electrocardiograph, and X-ray were performed, and chest computed tomography was performed if necessary.

### Endpoints and definitions

The primary endpoint was pulmonary infection during hospitalization, which was determined based on typical medical imaging and clinical signs, symptoms or relevant laboratory biomarkers (e.g., white blood cell count) by an experienced clinician [20]. For patients without sufficient evidence recorded for pulmonary infection diagnosis, the infection was determined using ICD-10-CM codes at discharge. The secondary endpoint was in-hospital major adverse cardiovascular events (MACEs), comprising all-cause mortality, stroke, target vessel revascularization, or recurrent MI.

### Statistical analyses

Continuous variables are shown as the mean ± SD or median (interquartile range) and were compared using independent *t*-test or Kruskal–Wallis test based on their distribution. Categorical variables are presented as numbers (percentages), and differences between groups were analyzed using Pearson’s chi-squared test or Fisher’s exact test. Variables with a statistical significance level of P < 0.10 on univariate logistic regression or with clinical importance, including age, sex, anemia, estimated glomerular filtration rate (eGFR), current smoker, DM, hypertension, chronic obstructive pulmonary disease, prior MI, prior PCI, prior stroke, transradial assessment, multi-vessel disease, and white blood cell count, were included and used for adjustment in the multivariate analysis. Receiver operating characteristic (ROC) curves were used to show the predictive value of the SHR. The best cutoff value and area under the ROC curve (AUC) were calculated. A cubic spine model was also performed to judge the effect of SHR on pulmonary infection. The logistic regression model was used to construct the cubic splines, and the knots were set at 0.75, 1, 1.25, 1.5, mainly based on the distribution of SHR. Statistical analysis was performed with SAS 9.4 (SAS Institute, Cary, NC). P < 0.05 were considered to indicate statistical significance, and all analyses were two-tailed.

## Results

### Baseline characteristics

**Fig. 1:**
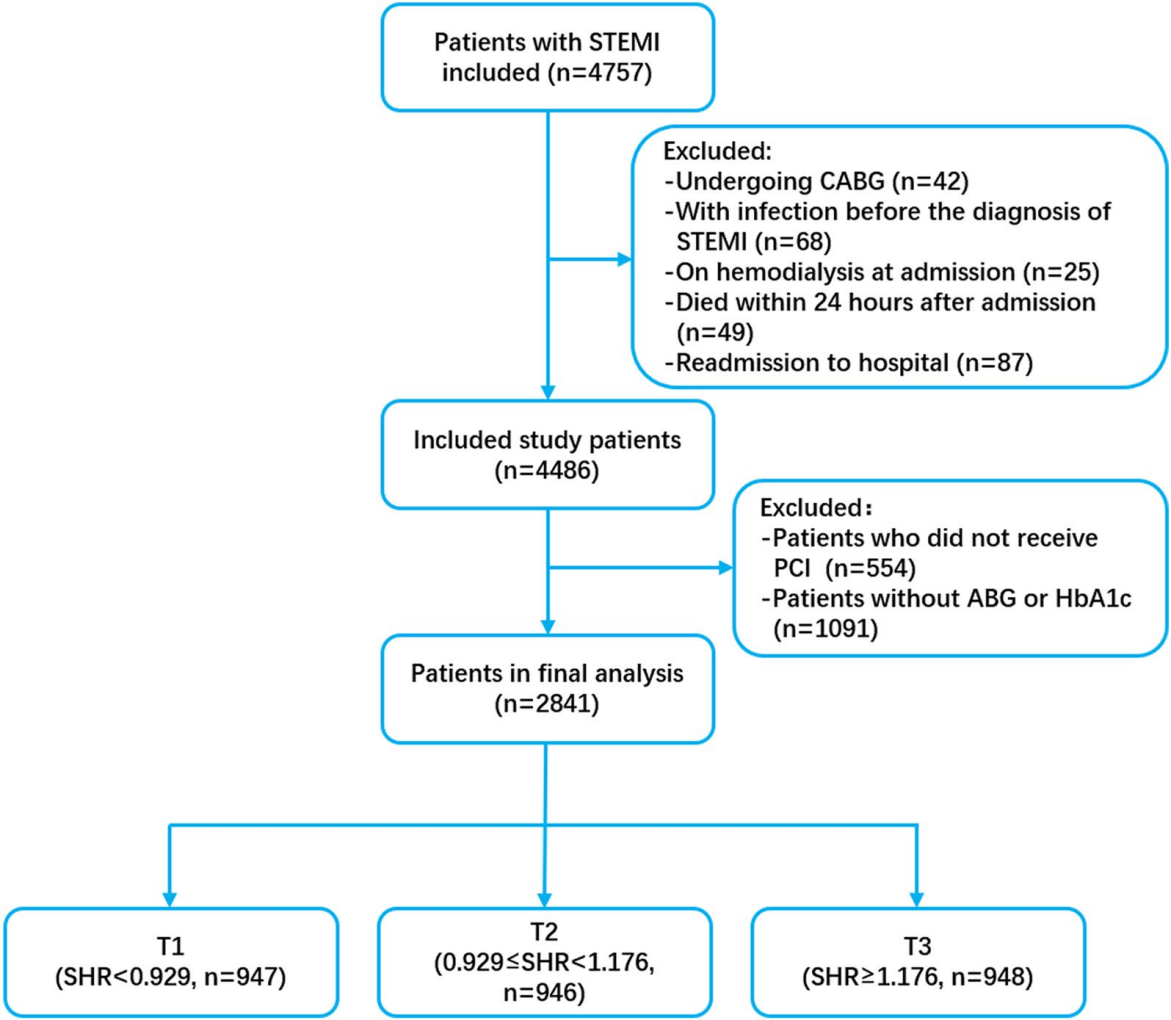
Flow chart of participants. *STEMI* ST-segment elevation myocardial infarction, *CABG* coronary artery bypass grafting, *PCI* percutaneous coronary intervention, *ABG* admission blood glucose, *HbA1c* glycosylated hemoglobin, *SHR* stress hyperglycemia ratio

From 2010 to 2020, 4,757 patients were diagnosed with STEMI. After excluding 42 patients undergoing coronary artery bypass grafting, 68 with infection before the diagnosis of STEMI, 25 on hemodialysis at admission, 49 subjects who died within 24 h after admission, 87 readmissions to hospital, 554 subjects who did not receive PCI and 1,091 without ABG or HbA1c, 2,841 patients were finally included. The study flow is shown in Fig. 1. The patients were divided into three groups according to the tertiles of SHR (n = 947, 946 and 948). The patients’ baseline characteristics are shown in Table 1.

**Table 1:** Comparison of baseline characteristics between groups stratified by tertile of SHR

Variables	Overall (n = 2841)	T1 (n = 947) SHR < 0.929	T2 (n = 946) 0.929 ≤ SHR < 1.176	T3 (n = 948) SHR ≥ 1.176	*P* value
Age, years	62.27 ± 12.18	61.67 ± 11.82	61.81 ± 12.53	63.32 ± 12.12	0.005
Male, n (%)	2347(82.6%)	810(85.5%)	794(83.9%)	743(78.4%)	< 0.001
SBP, mm Hg	122 ± 22	123 ± 21	122 ± 21	120 ± 22	0.019
DBP, mm Hg	74 ± 14	74 ± 13	74 ± 13	73 ± 13	0.045
Heart rate	80 ± 16	77 ± 14	80 ± 15	83 ± 17	< 0.001
Killip classification					< 0.001
I	1973 (69.5%)	692 (73.1%)	687 (72.7%)	594 (62.7%)	
II	586 (20.6%)	184 (19.4%)	186 (19.7%)	216 (22.8%)	
III	157 (5.5%)	40 (4.2%)	47 (5.0%)	70 (7.4%)	
IV	124 (4.4%)	31 (3.3%)	25 (2.6%)	68 (7.2%)	
Medical history, n (%)					
Current smoker	1179 (41.5%)	423 (44.7%)	409 (43.3%)	347 (36.6%)	< 0.001
Hypertension	1471(51.8%)	468 (49.4%)	488 (51.6%)	515 (54.3%)	0.101
Diabetes mellitus	882 (31.0%)	257 (27.1%)	245 (25.9%)	380 (40.1%)	< 0.001
Hyperlipidemia	361 (12.7%)	129 (13.6%)	127 (13.4%)	105 (11.1%)	0.180
Atrial fibrillation	91 (3.2%)	24 (2.5%)	37 (3.9%)	30 (3.2%)	0.235
COPD	65 (2.3%)	22 (2.3%)	26 (2.7%)	17 (1.8%)	0.379
Prior MI	698 (24.6%)	267 (28.2%)	208 (22.0%)	223 (23.5%)	0.005
Prior PCI	358 (12.6%)	141 (14.9%)	108 (11.4%)	109 (11.5%)	0.034
Prior stroke	202 (7.1%)	60 (6.3%)	61 (6.4%)	81 (8.5%)	0.109
Laboratory measurements					
WBC	11.66 ± 3.99	10.97 ± 3.60	11.77 ± 3.78	12.23 ± 4.45	< 0.001
Albumin	35.10 ± 4.43	35.15 ± 4.64	35.28 ± 4.26	34.86 ± 4.37	0.112
LVEF, %	51.26 ± 11.81	52.44 ± 11.54	51.77 ± 11.35	49.56 ± 12.32	< 0.001
Hemoglobin, g/L	134.19 ± 19.29	134.33 ± 18.90	135.64 ± 18.12	132.61 ± 20.67	0.003
Anemia	916 (32.3%)	302 (32.0%)	290 (30.7%)	324 (34.2%)	0.247
eGFR, mL/min	84.23 ± 31.60	85.21 ± 31.23	87.72 ± 30.78	79.75 ± 32.28	< 0.001
Admission blood glucose	8.81 ± 4.10	6.48 ± 2.16	8.20 ± 2.89	11.75 ± 4.80	< 0.001
HbA1c, %	6.10 (5.70–7.00)	6.20 (5.80–7.00)	6.00 (5.60–6.70)	6.10 (5.50–7.40)	< 0.001
Total cholesterol, mmol/L	4.89 ± 1.24	4.90 ± 1.23	4.92 ± 1.19	4.84 ± 1.31	0.372
LDL-C, mmol/L	3.21 ± 1.00	3.25 ± 0.98	3.23 ± 0.96	3.17 ± 1.06	0.193
Medication use					
Aspirin	2802 (98.6%)	941 (99.4%)	938 (99.2%)	923 (97.4%)	< 0.001
Clopidogrel	2496 (88.0%)	838 (88.5%)	814 (86.3%)	844 (89.1%)	0.145
Statins	2780 (97.9%)	928 (98.0%)	928 (98.1%)	924 (97.6%)	0.701
β-blockers	2324 (81.8%)	781 (82.5%)	775 (81.9%)	768 (81.1%)	0.738
ACEI/ARB	2254 (79.3%)	758 (80.0%)	745 (78.8%)	751 (79.2%)	0.782
Angiography					
Transradial assessment	2436 (85.9%)	851 (89.9%)	819 (86.8%)	766 (81.0%)	< 0.001
Multi-vessel stenosis, n (%)	2087 (73.5%)	707 (74.7%)	679 (71.8%)	701 (73.9%)	0.335
No. of stents	1.49 ± 2.04	1.54 ± 0.91	1.52 ± 3.31	1.41 ± 0.84	0.304
Length of stents, mm	37.43 ± 24.60	39.62 ± 26.13	36.22 ± 23.20	36.47 ± 24.27	0.004
Contrast volume, mL	115.66 ± 41.24	118.69 ± 44.78	112.34 ± 36.73	115.91 ± 41.57	0.005
Length of hospital stay, days	6.00 (5.00 ~ 9.00)	6.00 (5.00 ~ 8.00)	7.00 (6.00 ~ 8.00)	7.00 (6.00 ~ 11.00)	< 0.001

*SHR* stress hyperglycemia ratio, *SBP* systolic blood pressure, *DBP* diastolic blood pressure, *COPD* chronic obstructive pulmonary disease, *MI* myocardial infarction, *PCI* percutaneous coronary intervention, *WBC* white blood cell, *LVEF* left ventricular ejection fraction, *eGFR* estimated glomerular filtration rate, *HbA1c* glycosylated hemoglobin, *LDL-C* low-density lipoprotein cholesterol, *ACEI/ARB* angiotensin converting enzyme inhibitor/angiotensin receptor blocker

The mean age of the population was 62.27 ± 12.18 years, and 82.6% were male. 737 (83.6%) of the 882 DM diagnosis were based on medical history. For the remanent 145 newly diagnosis of DM, 15 (10.3%) participants were diagnosed with DM based on oral glucose tolerance test, 109 (75.2%) based both on glycemia and HbA1c, 13 (9.0%) based on HbA1c and 8 (5.5%) on glycemia only. As shown in Table 1, the higher SHR group was more likely to be older, female, has a higher Killip classification, and has a history of DM, but less likely to be current smokers. Meanwhile, the patients with lower SHR had a higher rate of prior MI and PCI, and in terms of in-hospital medication usage, a higher rate of aspirin use.

### SHR for pulmonary infection and in-hospital MACEs

**Fig. 2:**
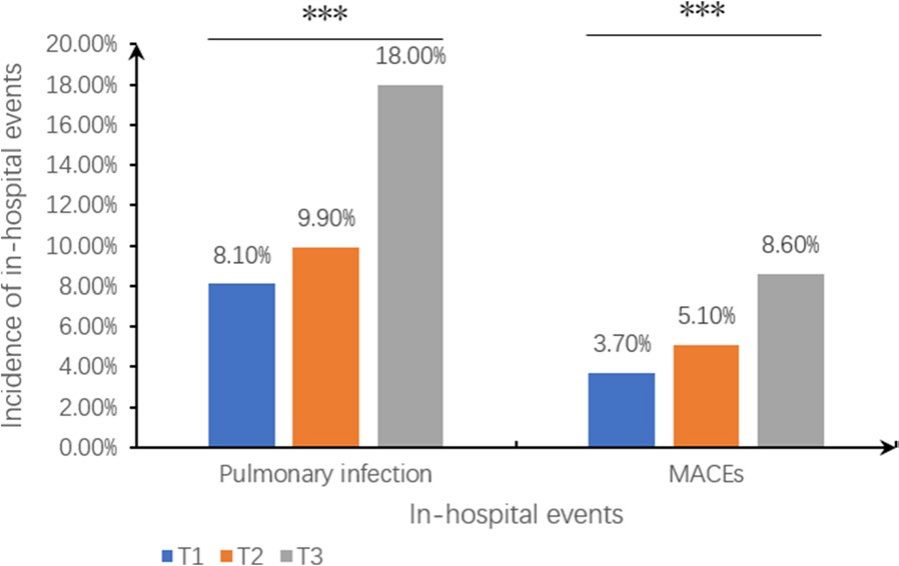
Incidence of in-hospital events in different SHR groups. ***P ≤ 0.001. *SHR* stress hyperglycemia ratio, *MACEs* major adverse cardiovascular events

During hospitalization, 323 (11.4%) patients developed the pulmonary infection and 165 (5.8%) suffered from in-hospital MACEs. The SHR was associated with pulmonary infection (8.1%, 9.9%, and 18.0%; P < 0.001) and in-hospital MACEs (3.7%, 5.1%, and 8.6%; P < 0.001), as shown in Fig. 2. In multivariate logistic regression analysis, SHR, as a continuous value, remained an independent risk factor significantly associated with pulmonary infection during hospitalization (adjusted odds ratio [OR] = 1.46, 95% confidence interval [CI] 1.06–2.02, P = 0.021) and in-hospital MACEs (adjusted OR = 1.67, 95% CI 1.17–2.39, P = 0.005) after adjusting for potential risk factors, including age, sex, anemia, eGFR, current smoker, DM and so on (Table 2). Taking SHR as a categorical value, the same results were found, in that a SHR more than 1.176 was a risk factor for a higher risk of pulmonary infection during hospitalization (adjusted OR = 1.75, 95% CI 1.25–2.46, P = 0.001) and in-hospital MACEs (adjusted OR = 1.68, 95% CI 1.09–2.60, P = 0.020) (Table 2).

**Table 2:** Multivariable logistic regression analysis for the SHR as categorical variable and continuous variable

Variables	Pulmonary infection			In-hospital MACEs		
	OR	95% CI	*P* value	OR	95% CI	*P* value
T1	Reference			Reference		
T2	1.21	0.84–1.74	0.296	1.32	0.83–2.11	0.238
T3	1.75	1.25–2.46	0.001	1.68	1.09–2.60	0.020
SHR*	1.46	1.06–2.02	0.021	1.67	1.17–2.39	0.005

Adjust for Age, Gender, eGFR, WBC, Anemia, Current smoker, DM, Hypertension, COPD, Prior MI, Prior PCI, Prior stroke, PCI assessment and Multi-vessel stenosis

*SHR* stress hyperglycemia ratio, *MACEs* major adverse cardiovascular events, *OR* odds ratio, *CI* confidence interval, *eGFR* estimated glomerular filtration rate, *WBC* white blood cell, *DM* Diabetes mellitus, *COPD* chronic obstructive pulmonary disease, *MI* myocardial infarction, *PCI* percutaneous coronary intervention

^*^SHR as continuous variable

**Fig. 3:**
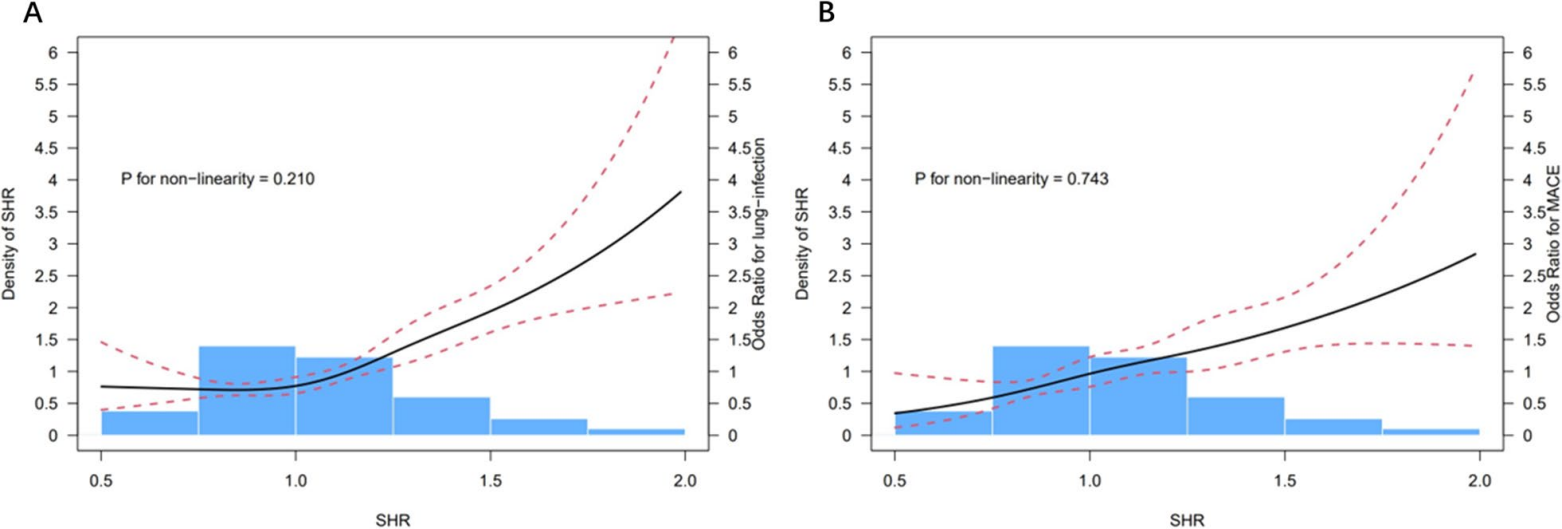
**A** Cubic spine models for the association between SHR and pulmonary infection. **B** Cubic spine models for the association between SHR and in-hospital MACEs. *SHR* stress hyperglycemia ratio, *MACEs* major adverse cardiovascular events

Cubic spline models demonstrated no significant non-linear relationship between SHR and pulmonary infection during hospitalization (P = 0.210) or MACEs (P = 0.743) (Fig. 3).

For the interaction between DM and the SHR, significant interactions were observed both for the pulmonary infection (interaction P = 0.012) and the in-hospital MACEs (interaction P = 0.009). For further analysis, we divided these patients into 6 groups, G1: without DM, SHR < 0.929 (T1); G2: without DM, 0.929 ≤ SHR < 1.176 (T2); G3: without DM, SHR ≥ 1.176 (T3); G4: with DM, T1; G5: with DM, T2 and G6: with DM, T3.

Multivariate logistic regression analysis demonstrated that DM was not significantly associated with the pulmonary infection (OR = 1.60, 95% CI 0.90–2.83, P = 0.110) nor the in-hospital MACEs (adjusted OR = 1.05, 95% CI 0.49–2.26, P = 0.898) for patients without stress hyperglycemia (T1). A SHR more than 1.176 (T3) was significantly associated with a higher risk of pulmonary infection during hospitalization (adjusted OR = 2.01, 95% CI 1.31–3.07, P = 0.001) but not for in-hospital MACEs (adjusted OR = 1.36, 95% CI 0.78–2.38, P = 0.276) for patients without DM.

A cumulative effect was observed for the in-hospital MACEs since DM with high SHR (T3) associated with higher risk than that of DM with low SHR (T1) and high SHR (T3) without DM. No cumulative effect was observed for the outcome of pulmonary infection. (Additional file 1: Table S1).

### Predictive value of the SHR for in-hospital events

**Fig. 4:**
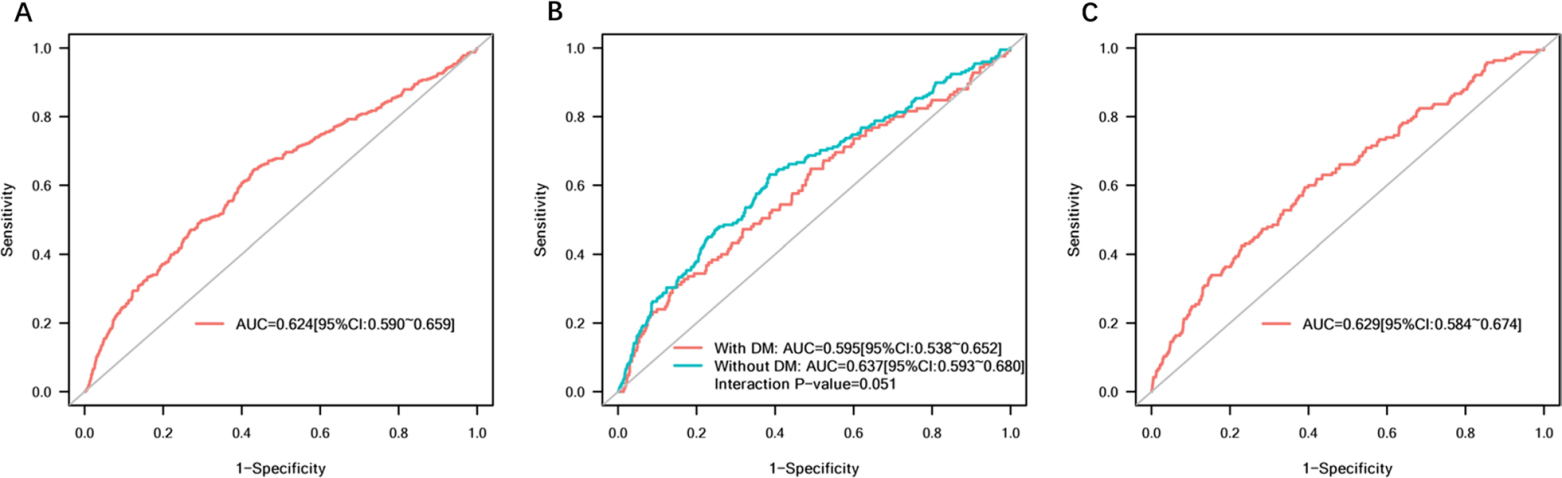
**A** ROC curve of SHR for pulmonary infection among STEMI patients. **B** ROC curve of SHR for pulmonary infection among STEMI patients with or without DM. **C** ROC curve of SHR for in-hospital MACEs among STEMI patients. *ROC curve* receiver operating characteristic curve, *AUC* area under the ROC curve, *DM* diabetes mellitus, *SHR* stress hyperglycemia ratio, *STEMI* ST-segment elevation myocardial infarction, *MACEs* major adverse cardiovascular events

The ROC curve of the SHR for pulmonary infection during hospitalization was drawn, and, as shown in Fig. 4A, had an AUC of 0.624 (95% CI 0.590–0.659). The best cutoff value of 1.073 was derived with a sensitivity of 64.7% and specificity of 56.8%. Subgroup analysis showed that the AUC was 0.595 (95% CI 0.538–0.652) and 0.637 (95% CI 0.593–0.680) for patients with and without DM, respectively (Fig. 4B). Additionally, an AUC of 0.629 (95% CI 0.584–0.674) was found for in-hospital MACEs (Fig. 4C).

## Discussion

To the best of our knowledge, this is the first study to establish a correlation between SHR and pulmonary infection during hospitalization among patients with STEMI undergoing PCI. Our main findings were that the SHR is independently correlated with pulmonary infection and MACEs in patients with STEMI during hospitalization.

### SIH and STEMI

SIH is a common status with a strong predictive value for worse clinical outcomes for patients with STEMI [5, 10, 11]. Several complex acute physiological changes, such as excessive gluconeogenesis, activation of the sympathetic-adrenergic-system, counter-regulatory hormones (e.g., catecholamine, cortisol), and proinflammatory cytokines (e.g., tumor necrosis factor-α ), play roles in the development of SIH and lead to a vicious cycle [8, 9, 21, 22]. This vicious cycle is also believed to contribute to worse outcomes for critically ill patients, including all-cause mortality and pneumonia [10, 11, 16, 23]. The relationship between SIH and the development of long- and short-term MACEs in patients with STEMI has been proven by prior studies [12, 14, 15]. In this research, we drew the same conclusion that SHR, reflecting SIH, is independently associated with the risk of MACEs during hospitalization. Despite firm evidence of the relationship between SIH and MACEs, the correlation between SIH and pulmonary infection during hospitalization for patients with STEMI remains unclear.

### SHR with pulmonary infection in patients with STEMI

As an important complication of STEMI, infection during hospitalization is associated with a worse clinical outcome [1–4], and several risk factors [24, 25] and scores [4, 26, 27] are utilized for infection prediction and prevention. Unfortunately, these predictive tools are largely complex or unable to be interfered.

In this research, we used the SHR, a simple and readily available parameter, on behalf of SIH, and found that it is independently associated with the risk of pulmonary infection during hospitalization for patients with STEMI. Although the underlying mechanism of this finding remains unclear, we hypothesize that the high pulmonary infection rate among high SHR patients with STEMI is attributed to the harmful effects of stress hyperglycemia. Glucose is considered a pro-inflammatory mediator, and elevation of blood glucose not only induces the generation of inflammatory cytokines, inflammatory processes, and insulin resistance [8, 9, 21], but also decreases vascular endothelial nitric oxide, which leads to vasoconstriction and low organ perfusion [28]. Moreover, glucose directly inhibits the function of T lymphocytes, immunoglobulin, and complement, disrupts the immune system, and further increases the risk of pulmonary infection [28]. In diabetic animal models, hyperglycemia can also lead to lower bacterial clearance and a higher rate of infection-related mortality [29–31]. Moreover, patients with high SHR have been found to be more likely to suffer from in-hospital MACEs [12, 32–34], which may increase the usage of invasive procedures, such as mechanical ventilation, thereby increasing the risk of pulmonary infection [35, 36]. This result provides a potential standard for admission blood glucose control for patients with STEMI during the acute phase, although further studies are needed.

This research found no significant non-linear relationship between the SHR and pulmonary infection or MACEs during hospitalization. Similarly, in the research of Yang et al. [34], although a J-shape relationship was confirmed between the SHR and major adverse cardiovascular and cerebrovascular events, MACE, cardiac death, and MI at a 2-year follow-up, neither a U nor J-shape relationship was found for in-hospital cardiac death and MI. These consistent findings highlight that the influence of the SHR on different outcomes in different periods may vary and have diverse mechanisms, which cannot yet be fully explained. Further studies are needed to clarify this phenomenon.

### SHR with infection in patients with STEMI with or without DM

The SHR was also found to be independently associated with the risk of pulmonary infection during hospitalization among patients with STEMI with or without DM. In the ROC curve of SHR for pulmonary infection, the AUC of patients without DM was slightly larger than that of patients with DM (P = 0.051). This tendency is consistent with previous studies that focused on other aspects of STEMI outcomes [12, 14]. As an explanation, chronic hyperglycemia of DM may induce antioxidant defenses and protect tissues and cells from the oxidative stress of acute hyperglycemia [37–39].

Although a strong association between SIH and STEMI prognosis has been declared, the optimal management for SIH is still debated. Moreover, clinical trials of SIH therapies have yielded conflicting results. Indeed, Kosiborod et al. [40] showed an association between glucose normalization and better survival in patients with acute myocardial infarction and SIH. In contrast, a meta-analysis of three clinical studies revealed few benefits of intensive blood glucose control, but an increased risk of serious hypoglycemia in patients with acute myocardial infarction and DM [41]. In this research, the SHR was proven to be independently associated with the risk of pulmonary infection and MACEs during hospitalization for patients with STEMI undergoing PCI. As the SHR, a better index of relative stress hyperglycemia, was shown to be a better predictor of worse outcomes, we propose that glycemic targets based on SHR instead of ABG may be applied to the management of acute hyperglycemia in further studies.

### Strengths and limitations

To the best of our knowledge, this is the first study to establish the correlation between SHR and pulmonary infection during hospitalization among patients with STEMI who underwent PCI. However, our study has several limitations. First, as this is an observational study, we could not adjust for all potential confounders. Second, only patients with STEMI in one center were included, which may limit the generalization of our conclusion. Third, blood glucose levels are prone to fluctuation, and the variation in durations from the onset of symptoms to the measurement of ABG and SHR may have impacted the results. Fourth, because of the small sample size of patients with STEMI without PCI, the impact of SHR on outcomes in these patients remained unclear. Therefore, further studies are needed to confirm the association between the SHR and pulmonary infection during hospitalization in patients with STEMI without PCI treatment.

## Conclusion

The SHR is independently associated with the risk of pulmonary infection during hospitalization and in-hospital MACEs for patients with STEMI who underwent PCI.

## Supplementary Information

**Additional file 1: Table S1.** Interaction and cumulative effects analysis between diabetes and SHR. **Figure S1.** Kaplan–Meier analyses for in-hospital pulmonary infection among the three groups. **Figure S2.** Kaplan–Meier analyses for in-hospital MACEs among the three groups. **Table S2.** Multivariable cox regression analysis for the SHR as categorical variable and continuous variable.

## Data Availability

The datasets used and analyzed during the present study are available from the corresponding author on reasonable request.

## Abbreviations

STEMI: ST-segment elevation myocardial infarction
SIH: Stress-induced hyperglycemia
ABG: Admission blood glucose
SHR: Stress hyperglycemia ratio
DM: Diabetes mellitus
PCI: Percutaneous coronary intervention
HbA1c: Glycosylated hemoglobin
MI: Myocardial infarction
MACEs: Major adverse cardiovascular events
eGFR: Estimated glomerular filtration rate
ROC: Receiver operating characteristic
AUC: Area under the ROC curve
OR: Adjusted odds ratio
CI: Confidence interval

## References

[1] Piccaro de Oliveira P, Gonzales V, Lopes RD, Schmidt MM, Garofallo S, Santos RP (2016). Serious infections among unselected patients with ST-elevation myocardial infarction treated with contemporary primary percutaneous coronary intervention. Am Heart J.

[2] Truffa AA, Granger CB, White KR, Newby LK, Mehta RH, Hochman JS (2012). Serious infection after acute myocardial infarction: incidence, clinical features, and outcomes. JACC Cardiovasc Interv.

[3] Putot A, Chague F, Manckoundia P, Cottin Y, Zeller M (2019). Post-infectious myocardial infarction: new insights for improved screening. J Clin Med.

[4] Liu Y, Dai Y, Chen J, Huang C, Duan C, Shao S (2020). Predictive value of the Canada Acute Coronary Syndrome risk score for post-acute myocardial infarction infection. Eur J Intern Med.

[5] Li M, Chen G, Feng Y, He X (2021). Stress induced hyperglycemia in the context of acute coronary syndrome: definitions, interventions, and underlying mechanisms. Front Cardiovasc Med.

[6] Kwoun MO, Ling PR, Lydon E, Imrich A, Qu Z, Palombo J (1997). Immunologic effects of acute hyperglycemia in nondiabetic rats. JPEN J Parenter Enteral Nutr.

[7] Fietsam R, Bassett J, Glover JL (1991). Complications of coronary artery surgery in diabetic patients. Am Surg.

[8] Morohoshi M, Fujisawa K, Uchimura I, Numano F (1996). Glucose-dependent interleukin 6 and tumor necrosis factor production by human peripheral blood monocytes in vitro. Diabetes.

[9] Esposito K, Nappo F, Marfella R, Giugliano G, Giugliano F, Ciotola M (2002). Inflammatory cytokine concentrations are acutely increased by hyperglycemia in humans: role of oxidative stress. Circulation.

[10] Timmer JR, Hoekstra M, Nijsten MW, van der Horst IC, Ottervanger JP, Slingerland RJ (2011). Prognostic value of admission glycosylated hemoglobin and glucose in nondiabetic patients with ST-segment-elevation myocardial infarction treated with percutaneous coronary intervention. Circulation.

[11] Kim EJ, Jeong MH, Kim JH, Ahn TH, Seung KB, Oh DJ (2017). Clinical impact of admission hyperglycemia on in-hospital mortality in acute myocardial infarction patients. Int J Cardiol.

[12] Gao S, Liu Q, Ding X, Chen H, Zhao X, Li H (2020). Predictive value of the acute-to-chronic glycemic ratio for in-hospital outcomes in patients with ST-segment elevation myocardial infarction undergoing percutaneous coronary intervention. Angiology.

[13] Roberts GW, Quinn SJ, Valentine N, Alhawassi T, O'Dea H, Stranks SN (2015). Relative hyperglycemia, a marker of critical illness: introducing the stress hyperglycemia ratio. J Clin Endocrinol Metab.

[14] Kojima T, Hikoso S, Nakatani D, Suna S, Dohi T, Mizuno H (2020). Impact of hyperglycemia on long-term outcome in patients with ST-segment elevation myocardial infarction. Am J Cardiol.

[15] Xu W, Yang YM, Zhu J, Wu S, Wang J, Zhang H (2022). Predictive value of the stress hyperglycemia ratio in patients with acute ST-segment elevation myocardial infarction: insights from a multi-center observational study. Cardiovasc Diabetol.

[16] Tao J, Hu Z, Lou F, Wu J, Wu Z, Yang S (2022). Higher stress hyperglycemia ratio is associated with a higher risk of stroke-associated pneumonia. Front Nutr.

[17] Alpert JS, Thygesen K, Antman E, Bassand JP. Myocardial infarction redefined--a consensus document of The Joint European Society of Cardiology/American College of Cardiology Committee for the redefinition of myocardial infarction. J Am Coll Cardiol. 2000;36(3):959-69. 10.1016/s0735-1097(00)00804-4 10987628

[18] American Diabetes A (2018). 2. Classification and diagnosis of diabetes: standards of medical care in diabetes-2018. Diabetes Care.

[19] Levine GN, Bates ER, Blankenship JC, Bailey SR, Bittl JA, Cercek B (2011). 2011ACCF/AHA/SCAI guideline for percutaneous coronary intervention. A report of the American College of Cardiology Foundation/American Heart Association task force on practice guidelines and the society for cardiovascular angiography and interventions. J Am Coll Cardiol.

[20] Torres A, Cilloniz C, Niederman MS, Menendez R, Chalmers JD, Wunderink RG (2021). Pneumonia. Nat Rev Dis Primers.

[21] Huberlant V, Preiser JC (2010). Year in review 2009: critical care–metabolism. Crit Care.

[22] Lazzeri C, Valente S, Chiostri M, Picariello C, Gensini GF (2012). Acute glucose dysmetabolism in the elderly with ST elevation myocardial infarction submitted to mechanical revascularization. Int J Cardiol.

[23] Bellaver P, Schaeffer AF, Dullius DP, Viana MV, Leitao CB, Rech TH (2019). Association of multiple glycemic parameters at intensive care unit admission with mortality and clinical outcomes in critically ill patients. Sci Rep.

[24] Dai Y, Wan X, Liu C, Duan C, Shao S, Chen H (2021). The predictive value of n-terminal probrain natriuretic peptide for infection in patients with acute myocardial infarction. Front Cardiovasc Med.

[25] Santos M, Oliveira M, Vieira S, Magalhaes R, Costa R, Brochado B (2021). Predictors and mid-term outcomes of nosocomial infection in ST-elevation myocardial infarction patients treated by primary angioplasty. Kardiol Pol.

[26] Liu Y, Wang L, Chen P, Dai Y, Lin Y, Chen W (2022). Risk estimation for infection in patients with ST-segment elevation myocardial infarction undergoing percutaneous coronary intervention: development and validation of a predictive score. Front Cardiovasc Med.

[27] Liu Y, Wang L, Chen W, Zeng L, Fan H, Duan C (2020). Validation and comparison of six risk scores for infection in patients with ST-segment elevation myocardial infarction undergoing percutaneous coronary intervention. Front Cardiovasc Med.

[28] Dandona P, Mohanty P, Chaudhuri A, Garg R, Aljada A (2005). Insulin infusion in acute illness. J Clin Invest.

[29] Eibl N, Spatz M, Fischer GF, Mayr WR, Samstag A, Wolf HM (2002). Impaired primary immune response in type-1 diabetes: results from a controlled vaccination study. Clin Immunol.

[30] Spatz M, Eibl N, Hink S, Wolf HM, Fischer GF, Mayr WR (2003). Impaired primary immune response in type-1 diabetes functional impairment at the level of APCs and T-cells. Cell Immunol.

[31] Edwards MS, Fuselier PA (1983). Enhanced susceptibility of mice with streptozotocin-induced diabetes to type II group B streptococcal infection. Infect Immun.

[32] Chen G, Li M, Wen X, Wang R, Zhou Y, Xue L (2021). Association between stress hyperglycemia ratio and in-hospital outcomes in elderly patients with acute myocardial infarction. Front Cardiovasc Med.

[33] Schmitz T, Freuer D, Harmel E, Heier M, Peters A, Linseisen J (2022). Prognostic value of stress hyperglycemia ratio on short- and long-term mortality after acute myocardial infarction. Acta Diabetol.

[34] Yang J, Zheng Y, Li C, Gao J, Meng X, Zhang K (2022). The impact of the stress hyperglycemia ratio on short-term and long-term poor prognosis in patients with acute coronary syndrome: insight from a large cohort study in Asia. Diabetes Care.

[35] Tobin M, Manthous C (2017). Mechanical ventilation. Am J Respir Crit Care Med.

[36] de Waha S, Desch S, Eitel I, Fuernau G, Lurz P, Sandri M (2014). Intra-aortic balloon counterpulsation - basic principles and clinical evidence. Vascul Pharmacol.

[37] Ceriello A, dello Russo P, Amstad P, Cerutti P (1996). High glucose induces antioxidant enzymes in human endothelial cells in culture. Evidence linking hyperglycemia and oxidative stress. Diabetes.

[38] Sechi LA, Ceriello A, Griffin CA, Catena C, Amstad P, Schambelan M (1997). Renal antioxidant enzyme mRNA levels are increased in rats with experimental diabetes mellitus. Diabetologia.

[39] Bellis A, Mauro C, Barbato E, Ceriello A, Cittadini A, Morisco C (2021). Stress-induced hyperglycaemia in non-diabetic patients with acute coronary syndrome: from molecular mechanisms to new therapeutic perspectives. Int J Mol Sci.

[40] Kosiborod M, Inzucchi SE, Krumholz HM, Masoudi FA, Goyal A, Xiao L (2009). Glucose normalization and outcomes in patients with acute myocardial infarction. Arch Intern Med.

[41] Chatterjee S, Sharma A, Lichstein E, Mukherjee D (2013). Intensive glucose control in diabetics with an acute myocardial infarction does not improve mortality and increases risk of hypoglycemia-a meta-regression analysis. Curr Vasc Pharmacol.

