# Supplementary material for: Positive association between stress hyperglycemia ratio and pulmonary infection in patients with ST-segment elevation myocardial infarction undergoing percutaneous coronary intervention
Source: Cardiovasc Diabetol. 2023 Mar 31;22:76. doi: 10.1186/s12933-023-01799-3 (PMC10067314; doi:10.1186/s12933-023-01799-3)
Supplement: Supplementary file 1 — Additional file 1: Table S1. Interaction and cumulative effects analysis between diabetes and SHR. Figure S1. Kaplan–Meier analyses for in-hospital pulmonary infection among the three groups. Figure S2. Kaplan–Meier analyses for in-hospital MACEs among the three groups. Table S2. Multivariable cox regression analysis for the SHR as categorical variable and continuous variable. [file 12933_2023_1799_MOESM1_ESM.docx]

**Additional file material**

**Positive association between stress hyperglycemia ratio and pulmonary infection in patients with ST-elevation myocardial infarction undergoing percutaneous coronary intervention**

**Additional file 1: Table S1-2, Figure S1-2**

**Additional file 1: Table S1 Interaction and cumulative effects analysis between diabetes and SHR**

| **Outcomes and factors** | **Univariate analysis** | | | **Multivariable analysis^$^** | | |
| --- | --- | --- | --- | --- | --- | --- |
|  | **OR** | **CI** | **P value** | **OR** | **CI** | **P value** |
| **Pulmonary** **Infection^*^** |  |  |  |  |  |  |
| T1 without diabetes | Reference | | | Reference | | |
| T2 without diabetes | 1.21 | 0.80~1.81 | 0.363 | 1.23 | 0.79~1.93 | 0.360 |
| T3 without diabetes | 2.79 | 1.93~4.06 | 0.000 | 2.01 | 1.31~3.07 | 0.001 |
| T1 with diabetes | 1.57 | 0.95~2.60 | 0.080 | 1.60 | 0.90~2.83 | 0.110 |
| T2 with diabetes | 2.12 | 1.32~3.41 | 0.002 | 1.87 | 1.10~3.16 | 0.021 |
| T3 with diabetes | 2.99 | 2.00~4.47 | 0.000 | 2.22 | 1.39~3.54 | 0.001 |
| **MACEs^#^** |  |  |  |  |  |  |
| T1 without diabetes | Reference | | | Reference | | |
| T2 without diabetes | 1.41 | 0.83~2.41 | 0.203 | 1.38 | 0.79~2.40 | 0.260 |
| T3 without diabetes | 1.99 | 1.18~3.36 | 0.010 | 1.36 | 0.78~2.38 | 0.276 |
| T1 with diabetes | 1.24 | 0.60~2.57 | 0.562 | 1.05 | 0.49~2.26 | 0.898 |
| T2 with diabetes | 1.68 | 0.86~3.31 | 0.132 | 1.26 | 0.62~2.56 | 0.525 |
| T3 with diabetes | 3.64 | 2.17~6.08 | 0.000 | 2.35 | 1.34~4.11 | 0.003 |

*Interaction between diabetes and SHR P=0.012；#Interaction between diabetes and SHR P=0.009

$Adjust for Age, Gender, eGFR, WBC, Anemia, Current smoker, Hypertension, COPD, Prior MI, Prior PCI, Prior stroke, PCI assessment, and Multi-vessel stenosis.

**Abbreviation:** SHR: stress hyperglycemia ratio; MACEs: major adverse cardiovascular events; OR: odds ratio; CI: confidence interval; eGFR: estimated glomerular filtration rate; WBC: white blood cell; COPD: chronic obstructive pulmonary disease; MI: myocardial infarction; PCI: percutaneous coronary intervention;

**Additional file 1: Figure S1.** **Kaplan-Meier analyses for in-hospital pulmonary infection among the three groups.**

**
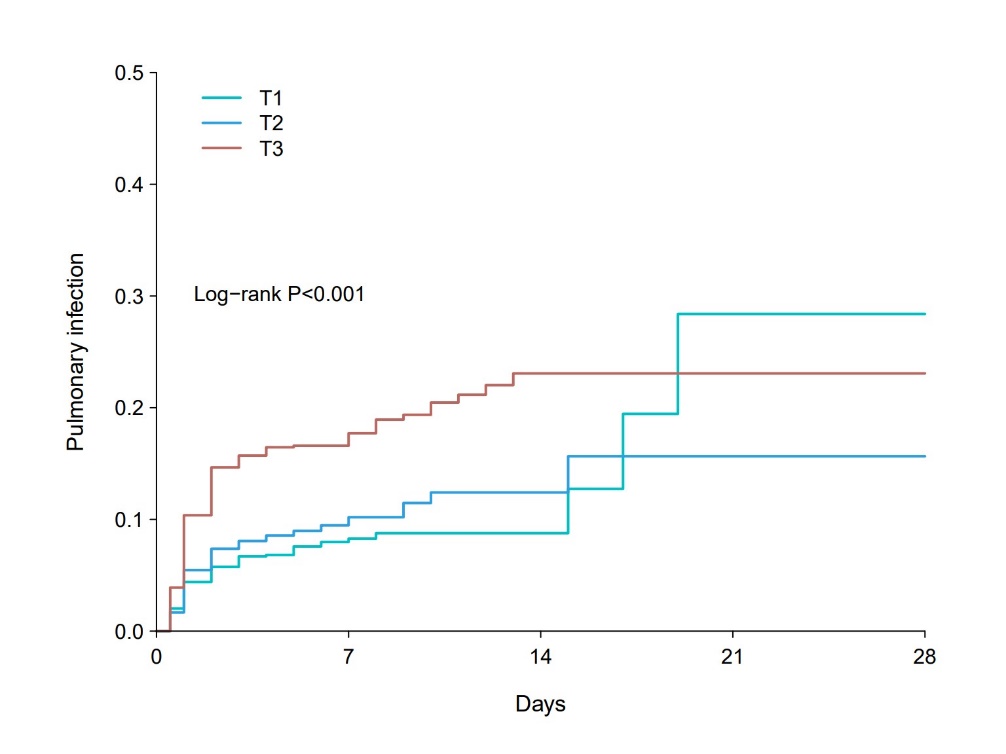
**

**Additional file 1: Figure S2. Kaplan-Meier analyses for in-hospital MACEs among the three groups.**


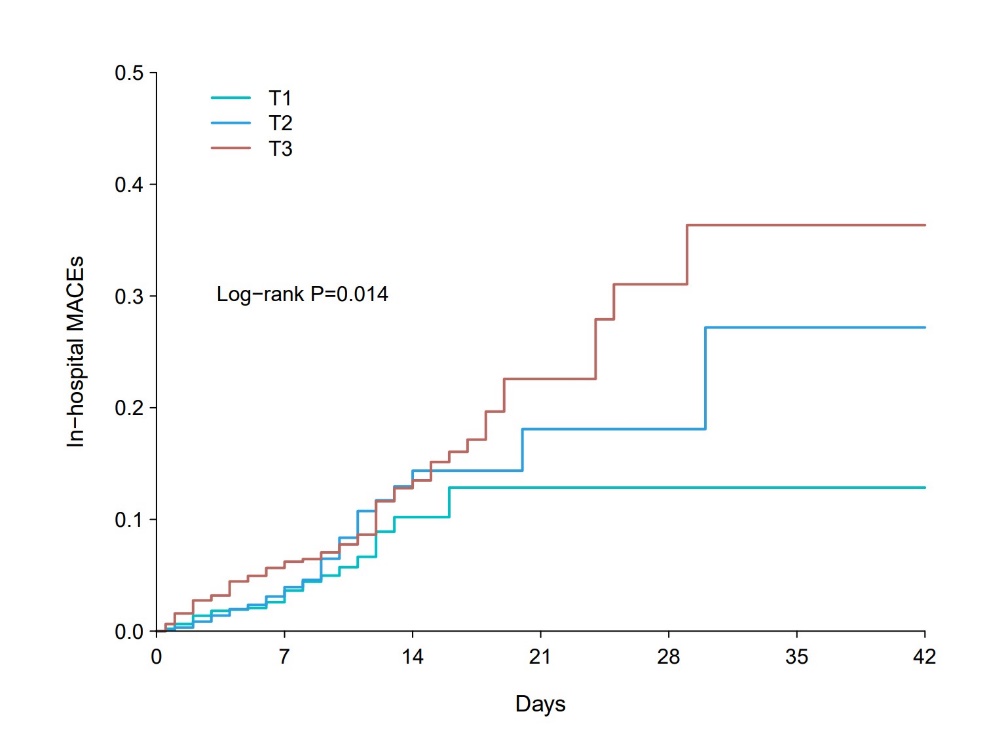


**Abbreviation:** MACEs: major adverse cardiovascular events.

**Additional file 1: Table S2. Multivariable cox regression analysis for the SHR as categorical variable and continuous variable.**

| **Variables** | **Pulmonary infection** | | |  | **In-hospital MACEs** | | |
| --- | --- | --- | --- | --- | --- | --- | --- |
|  | **HR** | **95% CI** | ***P* value** |  | **HR** | **95% CI** | ***P* value** |
| T1 | Reference | | |  | Reference | | |
| T2 | 1.17 | 0.85~1.60 | 0.337 |  | 1.25 | 0.81~1.95 | 0.315 |
| T3 | 1.53 | 1.14~2.05 | 0.004 |  | 1.46 | 0.97~2.21 | 0.07 |
| SHR* | 1.24 | 0.99~1.54 | 0.055 |  | 1.41 | 1.05~1.89 | 0.021 |

Adjust for Age, Gender, eGFR, WBC, Anemia, Current smoker, Diabetes mellitus, Hypertension, COPD, Prior MI, Prior PCI, Prior stroke, PCI assessment and Multi-vessel stenosis.

**Abbreviation:** SHR: stress hyperglycemia ratio; MACEs: major adverse cardiovascular events; HR: hazard ratio; CI: confidence interval; eGFR: estimated glomerular filtration rate; WBC: white blood cell; COPD: chronic obstructive pulmonary disease; MI: myocardial infarction; PCI: percutaneous coronary intervention.

***SHR as continuous variable.**
